# Oncology Workforce: More than Meets the Eye

**DOI:** 10.1007/s13187-025-02781-1

**Published:** 2025-11-15

**Authors:** Sierra Wood, Joseph Hines, Pauline Horton, Jessica Leib, Stacey Hembruff, Hope Krebill, Simon Craddock Lee, Lisa M. Harlan-Williams

**Affiliations:** 1 The University of Kansas Cancer Center, 3901 Rainbow Boulevard, Kansas City KS 66160, USA; 2 Department of Population Health, The University of Kansas Medical Center, Kansas City, USA; 3 Department of Cell Biology and Physiology, The University of Kansas Medical Center, Kansas City, USA

## Abstract

The current oncology workforce is struggling to keep up with demands for care. To address this shortage at The University of Kansas Cancer Center, we are educating the next generation about the breadth of careers at cancer centers. We describe the impetus for and process of developing an engaging infographic, “Charting the Course: Careers Along the Cancer Journey,” designed to resonate with high school students but which has also shown strong engagement and relevance among middle school students. The goal was to create a visual tool that not only illustrates the path of a cancer patient’s journey, but also highlights the various careers a cancer patient might encounter along the way. Ultimately, our aim is to build a comprehensive oncology workforce that is representative of the population we serve, thus educating the next generation of the oncology workforce and improving the health of our community at large.

## Introduction

The current oncology workforce is struggling to keep up with the demand for care [1]. The mission of The University of Kansas Cancer Center (KUCC) is to transform cancer research and the delivery of clinical care in our catchment area (the state of Kansas and the western edge of Missouri) and beyond. This landscape encompasses both urban and rural areas as well as under-resourced schools and well-resourced schools. An essential part of fulfilling our mission is training the next generation of the oncology workforce.

Job vacancy and turnover in the healthcare workforce is a growing concern in the United States; this is no exception in our catchment area. In Kansas and Missouri, the top four job vacancies in 2024 were magnetic resonance imaging technologist (20.6%), nurse assistant (16.1%), staff nurse (15.7%), and CT technologist (13.9%) [2, 3]. As a major employer in our area, KUCC has an important role in the community; however, many people still associate cancer centers with only doctors and nurses. Exposing the next generation to the multitude of careers at a cancer center supports our community as an employer of choice and an academic medical center.

**Fig. 1:**
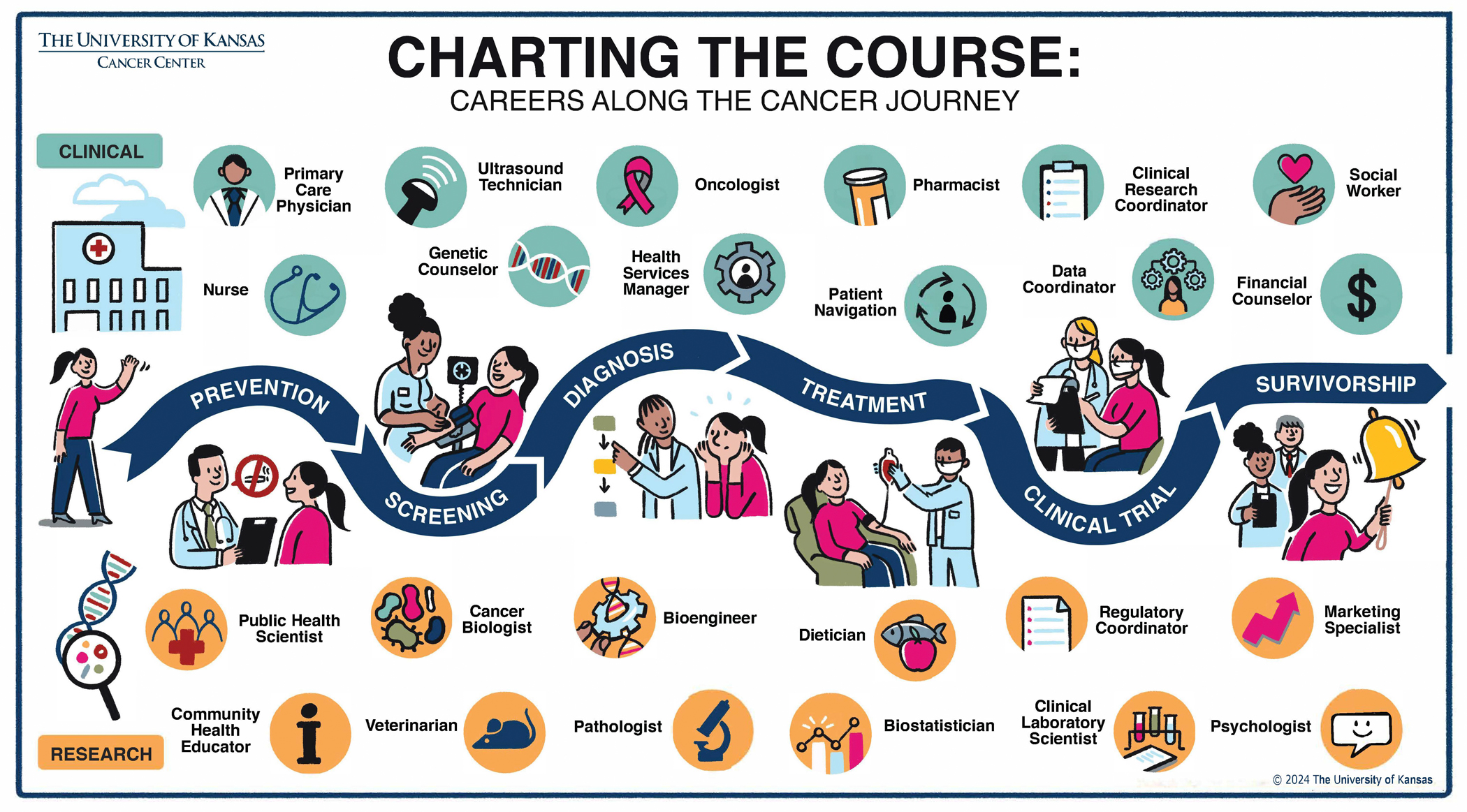
Oncology workforce infographic, charting the course: careers along the cancer journey. (Accessibility caption: A colorful journey map depicting a patient’s cancer journey from prevention, screening, and diagnosis to treatment, clinical trials, and survivorship. The map also shows the clinical and research careers they encounter along the way, such as nurse, oncologist, and pharmacist, and biologist, veterinarian, and biostatistician.)

With an understanding of employment as a social determinant of health [4], one upstream approach to improving cancer outcomes is to broaden students’ concept of the cancer workforce, and then, to encourage local recruitment into these roles. We therefore created an infographic that maps a cancer patient’s journey from screening to survivorship, highlighting the careers they may encounter along the way (Fig. 1).

## Process and Dissemination

This graphic created an accessible visual message about the variety of careers at a cancer center. In designing an infographic to resonate with all types of students, we raise awareness about the education and training opportunities available at every stage with the long-term goal of building a workforce that reflects our community.

We led outreach tours on campus and at local schools during which we held open-ended conversations with students about the variety of careers in the oncology workforce. Between August 2023 and September 2025, we visited 14 middle or high schools across the Kansas City metropolitan area, hosted nine groups of students, and engaged with over 300 students. We also engaged with 20 high school science teachers through two professional development workshops.

These conversations motivated us to standardize a way for students to think about the types of careers that are available, and we partnered with a graphic artist to develop a journey map. We were inspired by patient journey maps depicting the way a patient must navigate a healthcare system. Maps show both where someone has gone and where someone might go; our “Charting the Course” infographic shows the variety and necessity of careers along the journey. We also wanted to illustrate education and training opportunities, opening students’ minds to asking questions about education pathways to different careers.

The artist, along with our communications, community outreach, clinical trials, and education teams, contributed to the iterative, collaborative process. This group began by describing our institutional perspective on a patient’s cancer journey along the cancer care continuum, then dividing that journey into two parts: clinical care and research. Beginning with prevention, a person journeys through screening, early detection, diagnosis, treatment, perhaps a clinical trial, and hopefully enters survivorship. Along the way, patients interact with clinical professionals whose roles extend beyond the work of physicians and nurses, such as social workers. We discuss about the essential roles of nonclinical staff, like journalists, as well. We highlight how research is a fundamental aspect of high-quality clinical care; students are intrigued by veterinarians and dentists. Students visualize how research and clinical care how the two work together along a patient’s journey.

We have shared this infographic with middle and high school students and teachers and received positive feedback, responding to the clarity of the imagery and to the message conveyed. We encourage students to challenge us with careers that may not seem to fit into the cancer care continuum and to think critically about the operations of a health system and academic medical center – and the potential to pursue their own career goals within these institutions.

## Future Plans

The process of creating and disseminating this infographic has pushed us to think differently about educating the future oncology workforce – a workforce that must reflect and respond to the needs of the population we serve at multiple levels. It is important to raise awareness about the possibility of being a part of the oncology workforce without being a doctor or a nurse. As we have heard from our students, not everyone has the desire or the resources to pursue a long educational journey; however, the variety of careers in the oncology workforce mean that anyone – regardless of their background or interests – can have a role in caring for a cancer patient. Our refrain is that, when it comes to the oncology workforce, there is more than meets the eye.

Moving forward, we will continue to bring this infographic to schools and community programs across our catchment area. We will also expand our outreach to rural schools to tailor engagement to the rural context. Finally, we are exploring opportunities to adapt this tool to engage students in post-secondary settings, providing greater insights to inform their decisions as they explore career paths.

In the future, we intend to incorporate additional careers and generate an interactive infographic on the KUCC website. This interactive tool will link to our institution’s education and training programs. We also want to ensure we reach all populations of our catchment area. The best health outcomes are achieved when the clinical and research workforce reflects the patient populations [4, 5]. That begins with educating and training the next generation and emphasizing the relevance of our institution as an employer of choice for their future careers.
